# Evaluation of the prospective use of blood biomarkers for Alzheimer’s disease in primary and secondary care

**DOI:** 10.1002/alz.088404

**Published:** 2025-01-09

**Authors:** Sebastian Palmqvist, Gemma Salvadó, Niklas Mattsson‐Carlgren, Tim West, Ruben Smith, Rik Ossenkoppele, Suzanne E. Schindler, Kaj Blennow, Mark Monane, Philip B. Verghese, Joel B. Braunstein, Shorena Janelidze, Erik Stomrud, Pontus Tideman, Oskar Hansson

**Affiliations:** ^1^ Clinical Memory Research Unit, Department of Clinical Sciences Malmö, Faculty of Medicine, Lund University, Lund Sweden; ^2^ Clinical Memory Research Unit, Department of Clinical Sciences, Lund University, Lund Sweden; ^3^ Clinical Memory Research Unit, Lund University, Malmö Sweden; ^4^ C2N Diagnostics, LLC, Saint Louis, MO USA; ^5^ Knight Alzheimer Disease Research Center, St. Louis, MO USA; ^6^ Institute of Neuroscience and Physiology, Sahlgrenska Academy at the University of Gothenburg, Göteborg Sweden; ^7^ Clinical Memory Research Unit, Lund University, Lund Sweden

## Abstract

**Background:**

An accurate blood test for Alzheimer’s disease (AD) could streamline the diagnostic work‐up and treatment of AD. Our aim was to evaluate an AD blood test in primary and secondary care, using predefined biomarker cutoffs and prospective analyses of plasma samples.

**Method:**

940 prospective and unselected patients seeking medical evaluation for early cognitive symptoms were included. Plasma %p‐tau217 and Aβ42/40 was measured using mass spectrometry and the Amyloid Probability Score‐2 (APS2) combining these values was calculated. Predefined cutoffs were established in an independent training cohort and were applied to primary (n=307) and secondary care (n=300) cohorts comprised of patients undergoing cognitive evaluation where plasma samples were analyzed in single batches. The blood test was then evaluated prospectively in primary (n=100) and secondary (n=234) care patients where plasma samples were analyzed bi‐weekly. The main outcome was amyloid status as determined by cerebrospinal fluid AD biomarker positivity.

**Results:**

In primary care, 51% of patients were AD pathology positive and were diagnosed with the following: 25% subjective cognitive decline (SCD), 47% mild cognitive impairment (MCI), and 28% dementia. In secondary care 49% were AD pathology positive and diagnoses were 21% SCD, 43% MCI, and 36% dementia. Using single batch analyses in primary care, the AUC for APS2 was 0.97 (95% CI 0.95–0.99), PPV 91% (87–96%), and NPV 92% (87–96%); in secondary care, the AUC was 0.96 (0.94–0.98), PPV 88% (83–93%), and NPV 87% (82–93%). When samples were analyzed prospectively in primary care, the AUC was 0.97 (0.95–1.00), PPV 90% (81–99%), and NPV 90% (82–98%); in secondary care, the AUC was 0.97 (0.94–0.99), PPV 92% (86–97%), and NPV 89% (83–94%). Primary care physicians accurately identified AD in 58% of patients after a standard work‐up versus 89% (83–95%) for APS2 (p<0.001). Dementia experts had a diagnostic accuracy of 74% (69–80%) versus 90% (86–94%) for APS2 (p<0.001). At AAIC, we will also present prospective data using plasma p‐tau217 immunoassay‐based tests.

**Conclusions:**

Highly accurate AD blood tests might improve the diagnostic work‐up of individuals with cognitive symptoms in primary and secondary care. Future studies are needed to further evaluate these tests prospectively in primary care of other countries.

